# Differential Retinoic Acid Signaling in the Hippocampus of Aged Rats with and without Memory Impairment

**DOI:** 10.1523/ENEURO.0120-21.2021

**Published:** 2021-09-14

**Authors:** Marta U. Wołoszynowska-Fraser, Sharyn L. Rossi, Jeffrey M. Long, Peter J. McCaffery, Peter R. Rapp

**Affiliations:** 1 Laboratory of Behavioral Neuroscience, Neurocognitive Aging Section, National Institute on Aging, Baltimore, MD 21224; 2 Institute of Medical Science, School of Medical Sciences, University of Aberdeen, Foresterhill, Aberdeen AB25 2ZD, Scotland, United Kingdom

**Keywords:** aging, hippocampus, memory, retinoic acid, spatial, vitamin A

## Abstract

Retinoic acid (RA), a metabolite of vitamin A, has many physiological functions, and mounting evidence points to important roles in cognition. *In vitro* experiments indicate that RA is involved in homeostatic synaptic scaling in the hippocampus, which supports overall network stability during learning. It has been previously determined that disrupted RA signaling in the hippocampus causes deterioration of memory, that RA signaling declines with age in brain, and that application of RA reverses this decline. Here, we explore whether RA signaling is altered in an animal model of neurocognitive aging. We used a Morris water maze protocol to study cognitive decline in aged rats, which assesses hippocampus-dependent spatial memory and reveals substantial interindividual differences in aged animals. Aged unimpaired (AU) rats perform on par with young (Y), while aged impaired (AI) animals exhibit spatial memory deficits. We show that the major substrate for RA, retinol binding protein 4 (RBP4), is decreased in AU rats, and retinol cell surface receptor declines with chronological age. Other affected components of RA signaling include selective increases in AI animals in hippocampal synthesis (RALDH1) and catabolism of RA (CYP26B1), RA receptor α, the RA regulated ionotropic glutamate receptor (GluR1), as well as fragile X mental retardation protein (FMRP). The results support the conclusion that, surprisingly, increased RA signaling in the aged hippocampus is associated with poor cognitive outcome.

## Significance Statement

Growing evidence indicates that retinoic acid (RA) function extends well beyond metabolic control and includes the regulation of memory-related synaptic plasticity. Here, we explore whether RA signaling is altered in an animal model of neurocognitive aging. We show that in fact RA function is altered at nearly all levels examined, and these results are unrelated to metabolic aging. Overall, the net effect points in the direction of increased RA signaling in impaired aged animals, which may contribute to disruption in excitation/inhibition balance, a prominent feature of age-related cognitive impairment and suspected early event in the pathogenesis of Alzheimer’s disease.

## Introduction

Circulating levels of retinoic acid (RA), a metabolite of vitamin A (retinol), are dependent on dietary availability because animals are unable to synthesize retinol *de novo*. Dietary sources can be from plants in the form of carotenoids or animal sources (retinyl esters; Blomhoff and Blomhoff, 2006). RA has many physiological functions, including control of neuronal differentiation during development, and modulation of neuronal plasticity and neurogenesis in the adult hippocampus (Maden, 2007; Nomoto et al., 2012; Chen et al., 2014). A potential role in memory processes is emerging and RA supplementation as a potential intervention for successful cognitive aging has received preliminary support (Mingaud et al., 2008; Dumetz et al., 2020). Complementing these findings, retinol deficiency during adolescence causes memory impairments comparable to those seen in aged rodents, and vitamin A supplementation can reverse these deficits (Etchamendy et al., 2001; Bonnet et al., 2008). The age-related reduction of plasma retinol binding protein (RBP; Kocełak et al., 2018), and retinol (Van Der Loo et al., 2004), as well as decreased vitamin A metabolism (Touyarot et al., 2013), suggest an overall decrease in RA signaling in aging (Enderlin et al., 1997; Etchamendy et al., 2003; Das et al., 2014). A global diminishment in RA functions may link metabolic aging and neurobiological mechanisms responsible for age-associated cognitive decline.

In the blood, retinol circulates freely or bound to RBP4, which is carried by transthyretin (TTR). TTR allows stable transport of RBP4 bound retinol and prevents RBP4 filtration and degradation by the kidney (Kanai et al., 1968; Palha, 2002; Vieira and Saraiva, 2014). Circulating retinol enters the cell via stimulated by RA-6 (STRA6) receptor or, because of its lipophilic properties, via cell membrane diffusion (Napoli, 2012; O’Byrne and Blaner, 2013). Inside the cell, retinol binds to the cellular RBPs and is further metabolized to RA. The last step of RA synthesis is catalyzed by the retinaldehyde dehydrogenase enzymes (RALDHs). RA can exhibit genomic or non-genomic functions, via binding to RA receptors (RARs and RXRs), diffuse to neighboring cells, or be catabolized by the cytochrome p450 family enzymes (CYP26s; Chen and Napoli, 2008; Chen et al., 2008; Shearer et al., 2012).

The growth, development, and ability of neurons to adapt to a changing environment are crucial for normal cognition. Mounting evidence points to the involvement of RA in memory formation. RA is involved in homeostatic synaptic scaling in the hippocampus, which maintains neuronal network stability in the face of learning-induced changes in synaptic strength (Groth and Tsien, 2008). This modulation is mediated through RA binding to its receptor α (RARα), which acts as an RNA-binding granule (Maghsoodi et al., 2008), promoting the dissociation of ionotropic glutamate receptor (GluR1) mRNA bound to RARα, making it available for translation. The fragile X mental retardation protein (FMRP) is required for the translation of GluR1, which results in an increase of dendritic synthesis of GluR1 and synaptic strength (Aoto et al., 2008; Soden and Chen, 2010). The expression of glutamate receptors and glutamate uptake decline with age, potentially contributing to memory decline (Segovia et al., 2001; Yang et al., 2015).

To examine the link between hippocampal RA signaling and neurocognitive aging, we used a well-established animal model of age-related cognitive decline. In this model, the hippocampus in aged rats with spatial memory deficits displays a complex constellation of changes relative to younger animals and age-matched subjects with intact memory, including a decrease in the number of inhibitory somatostatin neurons in the dentate gyrus, increased basal Arc protein expression but diminished behavioral induction in the pyramidal cell fields, as well as pyramidal neuron hyperactivity in the CA3 region (Wilson et al., 2005; Spiegel et al., 2013; Fletcher et al., 2014). Here, we measured plasma RBP4 and protein levels of hippocampal STRA6 receptor, RA synthesizing and catabolizing enzymes, RARα, FMRP, and GluR1 in young (Y) rats and aged animals with and without memory impairment.

## Materials and Methods

### Animals

Y (six months; *n* = 16) and aged (24 months; *n* = 32) male Long–Evans rats (Charles River Laboratories) were single housed in a climate-controlled vivarium, on a 12/12 h light/dark cycle. Animals had *ad libitum* access to food (Teklad Global 18% protein extruded rodent diet with vitamin A acetate, 30 IU/g, 1 IU = 0.3 μg retinol) and water. Rats were screened for health conditions, including skin conditions incompatible with water maze exposure, cataracts and tumors. Only healthy animals were used.

### Ethical statement

This study was conducted in accordance with the recommendations in the *Guide for the Care and Use of Laboratory Animals* of the National Institutes of Health. The protocol was approved by the Animal Care and Use Committee of the National Institute on Aging (ASP number LBN-407-2020).

### Spatial learning and memory/background behavioral characterization

Hippocampal-dependent spatial learning and memory were assessed using a Morris water maze protocol, optimized for documenting individual differences in aging (Gallagher et al., 1993). Briefly, animals were trained to find the location of a hidden platform, three trials per day over eight consecutive days. Probe trials were interpolated throughout training (one the last trial of every other day), to record spatial bias for the location of the platform. For the probes, the platform was unavailable for escape during the first 30 s of the trial. A learning index (LI) score for each animal was calculated based on average proximity (in centimeters) to the hidden escape location across the last three probe trials. Lower LI scores indicate better search accuracy focused on the escape location. Aged animals that performed on par with Y were classified as aged unimpaired (AU), while aged animals that performed above an LI cutoff based on Y were classified as aged impaired (AI). The cut off (LI of 240) is based on the normative distribution of scores for many hundreds of Y rats in previous research (Gallagher et al., 1993; Rapp and Gallagher, 1996; Maei et al., 2009; Haberman et al., 2012; Tomás Pereira and Burwell, 2015).

### Tissue collection

Euthanasia for postmortem analysis occurred in the morning/early afternoon no sooner than two weeks after completing water maze training, to minimize the influence of behavioral testing on the RA measures of interest. Animals were disoriented or briefly anaesthetized using Isoflurane and decapitated. Trunk blood was collected in heparinized tubes, which were slowly inverted multiple times and placed on wet ice. Blood was transferred to Eppendorf tubes and spun at 1500 G for 20 min at 4°C. Supernatant (plasma) was then transferred to a separate tube and both were kept at −80°C. Brains were extracted, and hippocampi were rapidly dissected over ice, snap frozen on dry ice, and stored at −80°C until required.

### ELISA

RBP4 ELISA was performed using a rat RBP4 kit (Abcam, ab203362). Plasma samples were diluted to 1:500,000, before performing the assay. A standard curve was created using stock RBP supplied and results were obtained by measuring the absorption at 450 nm.

### Plasma glucose, triglyceride, and creatinine assays

Plasma glucose levels were measured with an assay kit (Abcam, ab65333). Plasma samples were first deproteinized with 10-kDa spin columns (Abcam, ab93349). A standard curve was generated using stock glucose provided. The assay was run per manufacturer instructions and the absorption was measured at 570 nm. Triglyceride content was tested using an assay kit (Biovision, #622). Manufacturer’s instructions were followed to obtain the standard curve from the triglyceride stock provided, the results were obtained by measuring the absorption at 570 nm, and plasma triglyceride content was calculated. An assay kit was used to determine plasma creatinine content (Biovision, #625). Deproteinized plasma samples were tested. Manufacturer instructions were followed, and stock creatinine was used to create a standard curve and absorption was read at 570 nm.

### Protein extraction and quantification

Proteins from one hippocampus per animal (Y *n* = 16, aged *n* = 32) were extracted using tissue protein extraction reagent (T-PER; Thermo Fisher Scientific) with HALT protease inhibitor cocktail (100×; Thermo Fisher Scientific), homogenized, sonicated, spun down for 20 min (13,200 rpm at 4°C) and supernatant collected. Protein content in the supernatant was measured using Pierce’s bicinchoninic acid (BCA) assay (Thermo Fisher Scientific). Proteins were made into stock solution of 5 mg/ml.

### Synaptosome preparation

Synaptic protein extraction reagent (Syn-PER; Thermo Fisher Scientific) was used to isolate synaptosomes, which contain key presynaptic and postsynaptic proteins. Briefly, 10 ml of Syn-PER was added to each milligram of hippocampal tissue, which was then homogenized using Dounce homogenizer. Samples were then centrifuged at 3600 rpm for 10 min at 4°C. Supernatant was placed in a fresh tube and centrifuged again at 12 700 rpm for 20 min at 4°C. Supernatant was then removed, and the pellet resuspended in Syn-PER (2 ml/g of tissue). Again, protein content was measured by BCA assay and made into 5 mg/ml stock solution.

### Western blotting

Samples (25 mg of protein per well/lane) were separated by SDS-PAGE Bis-Tris gel (Invitrogen) and MES-SDS buffer (Invitrogen). All groups (Y, AU, and AI) were represented within each gel. Following separation, proteins were transferred onto PVDF membranes (Invitrogen) using an iBlot dry blotting system (Invitrogen). The antibodies were diluted in blocking solution (2% ELC advance blocking agent; GE Healthcare) in wash buffer (TBS and 0.1% Tween 20) and membranes were incubated overnight at 4°C. Immunoreactivity was detected by application of conjugated secondary antibodies (Alexa Fluor 488; Alexa Fluor 546; CY5; Jackson ImmunoResearch). The immunoblots were scanned on a Sapphire imager (Azure Biosystems) at 100-mm resolution and quantified using ImageJ software (National Institutes of Health). Glyceraldehyde 3-phosphate dehydrogenase (GAPDH) or β-actin were used as loading controls and all results were normalized to the expression of these proteins.

### Primary antibodies

The primary antibodies and their dilutions are listed in Table 1.

**Table 1:** Primary antibodies used for the Western blotting (WB) with corresponding dilutions

Antibody name	Host	WB dilution	Predicted molecular weight	Supplier	Catalog number
Anti-RALDH1	Goat	1:3000	55 kDa	Abcam	ab9883
Anti-RALDH3	Rabbit	1:1000	57 kDa	Abcam	ab129815
Anti-β-actin	Mouse	1:30,000	45 kDa	BioVision Incorporated	3598
Anti-CYP26A1	Mouse	1:1000	49 kDa	Santa Cruz Biotechnology	sc-53618
Anti-CYP26B1	Rabbit	1:1000	58–60 kDa	ProteinTech Antibodies	21555-1-AP
Anti-FMRP	Rabbit	1:1000	80 kDa	Abcam	ab17722
Anti-GAPDH	Rabbit	1:10,000	37 kDa	Santa Cruz Biotechnology	sc-25778
Anti-GluR1	Rabbit	1:500 1:250 (s)	106 kDa	ThermoFisher Scientific	PA1-46151
Anti-PSD95	Rabbit	1:500	95 kDa	Millipore	AB9708
Anti-RARα	Goat	1:2000 1:1000 (s)	51 kDa	Abcam	ab28767
Anti-Stra6	Rabbit	1:1000	95 kDa	ProteinTech Antibodies	22001-1-AP

(s), synaptosome preparation

### Statistical analysis

Statistical analysis was performed using Prism 7.0 (GraphPad Software). Unless otherwise stated, unpaired two-tailed Student’s *t* test was used when comparing two groups, while two-way ANOVA with Tukey’s multiple comparison test was applied to multiple group analysis. Pearson r correlation coefficients were calculated to investigate associations between variables of interest. Values of *p* < 0.05 were considered statistically significant. An observation was considered an outlier if its value was >2 SDs from the mean. This was the case for only one Y animal, which was excluded from the RALDH1 analysis.

## Results

### Aged rats display increased individual differences in spatial memory

**Figure 1.**
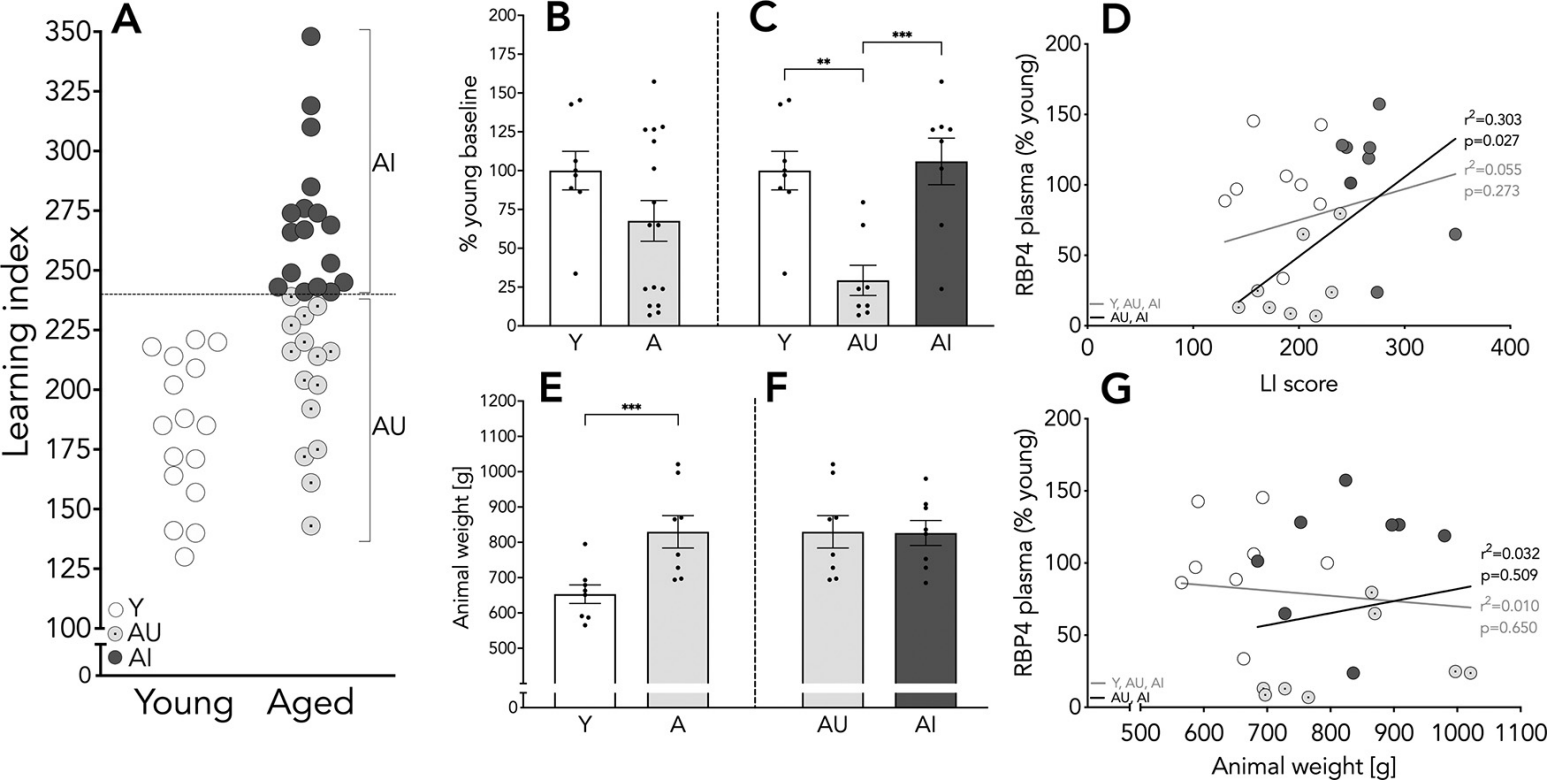
Spatial memory performance, animal weight, and plasma RBP4 levels. LI scores for individual Y and aged animals (***A***); plasma RBP4 levels presented as % of Y baseline (***B***, ***C***); correlation of plasma RBP4 and LI score (***D***); mean body weights of animals tested. Note that body weight did not differ among the aged rats (***E***, ***F***); correlation of plasma RBP4 and body weight (***G***). Results shown as bars with individual animal data plotted. Statistical analysis, unpaired two-tailed Student’s *t* test (***B***, ***E***, ***F***), one-way ANOVA, with Tukey’s multiple comparisons test (***C***), and linear regression (***D***, ***G***; all animals, gray line; aged animals, black line); **p < 0.01, ***p < 0.001. Y *n* = 16 and aged *n* = 32 (AU *n* = 15, AI *n* = 17; panel ***A***); Y *n* = 8 and aged *n* = 16 (AU *n* = 8, AI *n* = 8; panels ***B–G***). Error bars represent SEM.

Spatial memory was assessed in the Morris water maze using a protocol optimized for the study of cognitive aging (Gallagher et al., 1993). Over the course of testing rats were given probe trials to assess spatial bias for the platform location, and LI scores were calculated for each animal. As predicted, aged animals, on average displayed higher LI scores [Y mean = 182.3 (*n* = 16), aged mean = 239.1 (*n* = 32), *t*_(46)_ = 4.537, *p* < 0.0001; Fig. 1*A*]. Consistent with many earlier studies (Lee et al., 2005; Castellano et al., 2012; Fletcher et al., 2014; Myrum et al., 2019), the aged group exhibited substantially increased variability such that some rats performed on par with Y, while others performed well outside the normal range. Aged animals with LI scores below 240 (*n* = 15) were classified as AU, and rats that scored above 240 (*n* = 17) were operationally defined AI. This animal model provides an opportunity to test for chronological age effects (Y vs aged), while also allowing the study of mechanisms of cognitive aging, by comparing the Y, AU, and AI, and exploring potential linear correlations between LI scores and RA signaling factors in the same subjects.

### Plasma RBP4 is reduced in aged animals without memory impairment

Retinol can circulate free in blood or bound to RBP4/TTR complex. We tested plasma RBP4 concentration, which is indicative of retinol availability. We found no differences in plasma RBP4 between the Y and aged groups (*t*_(22)_ = 1.568, *p* = 0.131; Fig. 1*B*). However, RBP4 levels were significantly lower in AU compared with Y and AI rats (*F*_(2,21)_ = 11.48, *p* = 0.0004; Y vs AU *p* = 0.0019; AU vs AI *p* = 0.0009; Fig. 1*C*). Additionally, levels of circulating RBP strongly correlated with spatial memory performance among the aged rats such that subjects with higher RBP4 levels displayed worse spatial memory (*n* = 16, *r*^2^ = 0.303, *p* = 0.027; Fig. 1*D*, black line). The correlation was not significant when the data for Y rats were included in the analysis (*n* = 24, *r*^2^ = 0.055, *p* = 0.273; Fig. 1*D*, gray line), suggesting that coupling between RBP4 availability and hippocampal memory function emerges specifically in relation to cognitive aging.

Although the liver is the major peripheral source of RBP4, it is also released by adipocytes (Thompson et al., 2017), and elevated RBP4 levels have been reported in obese and diabetic individuals (Yang et al., 2005; Esteve et al., 2009). Since aged rats are heavier than Y adults (Y mean = 653 g, aged mean = 828 g, *t*_(22)_ = 3.979, *p* = 0.0006; Fig. 1*E*) we examined whether RBP4 levels correlate with body weight. In the aged group there was no difference in body weight between AU and AI rats (*t*_(14)_ = 0.06, *p* = 0.95; Fig. 1*F*). RBP4 levels were unrelated to body weight in Y and aged animals considered together (*n* = 24, *r*^2^ = 0.01, *p* = 0.65; Fig. 1*G*, gray line), and no correlation between body weight and RBP levels was detected when AU and AI rats were considered alone (*n* = 16, *r*^2^ = 0.032, *p* = 0.032; Fig. 1*G*, black line). These results indicate that RBP4 levels in aging are more tightly linked with individual differences in cognitive outcome than with the effects of chronological age, per se.

### Aged Long–Evans rats do not display metabolic syndrome

**Figure 2.**
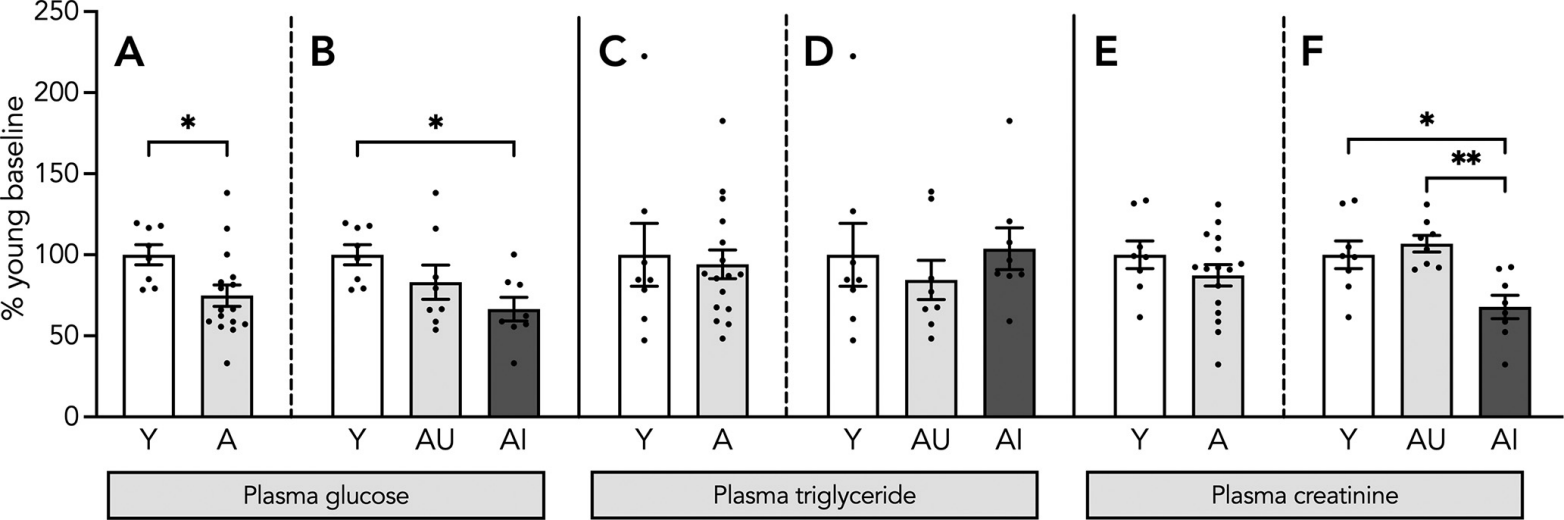
Plasma glucose, triglyceride, and creatinine levels. Levels of plasma glucose (***A***, ***B***) triglyceride (***C***, ***D***), and creatinine (***E***, ***F***) in the Y, aged, AU, and AI groups. Results shown as bars with individual animal data plotted. Statistical analysis, unpaired two-tailed Student’s *t* test (***A***, ***C***, ***E***), and one-way ANOVA, with Tukey’s multiple comparisons test (***B***, ***D***, ***F***); *p < 0.05, **p < 0.01. Y *n* = 8 and aged *n* = 16 (AU *n* = 8, AI *n* = 8; all panels). Error bars represent SEM.

Plasma glucose, triglyceride and creatinine content were measured to determine whether aged animals display metabolic symptoms that might affect RBP4 levels. Plasma glucose was significantly lower in aged animals compared with Y (*t*_(22)_ = 2.445, *p* = 0.023; Fig. 2*A*). This difference was especially prominent when AI rats were compared with Y (*F*_(2,21)_ = 4.155, *p* = 0.03; Y vs AI, *p* = 0.023; Fig. 2*B*), but not with AU (*p* = 0.34). Thus, despite substantially increased weight, pancreatic function seems to keep glucose levels on par with, or lower than, in Y rats compared with AU and AI animals. Plasma triglyceride content was measured to test liver function, and the results showed no difference between Y and aged animals (*t*_(22)_ = 0.319, *p* = 0.75; Fig. 2*C*), or when aged rats were split depending on their cognitive profile (*F*_(2,21)_ = 0.454, *p* = 0.64; Fig. 2*D*). Kidney function was assessed indirectly via plasma creatinine levels. Subjects with chronic kidney disease often display increased circulating RBP4 (Kocełak et al., 2018; Xun et al., 2018). We saw no differences in plasma creatinine levels between Y and aged rats (*t*_(22)_ = 1.133, *p* = 0.27; Fig. 2*E*). However, we found significantly lower plasma creatinine in AI group compared with Y and AU rats (*F*_(2,21)_ = 8.59, *p* = 0.002; Y vs AI, *p* = 0.0116; AU vs AI, *p* = 0.002; Fig. 2*F*). These data taken together indicate that the RBP4 results in aged rats are not a secondary consequence metabolic disease.

### Hippocampal STRA6 receptor expression is reduced in aged animals

**Figure 3.**
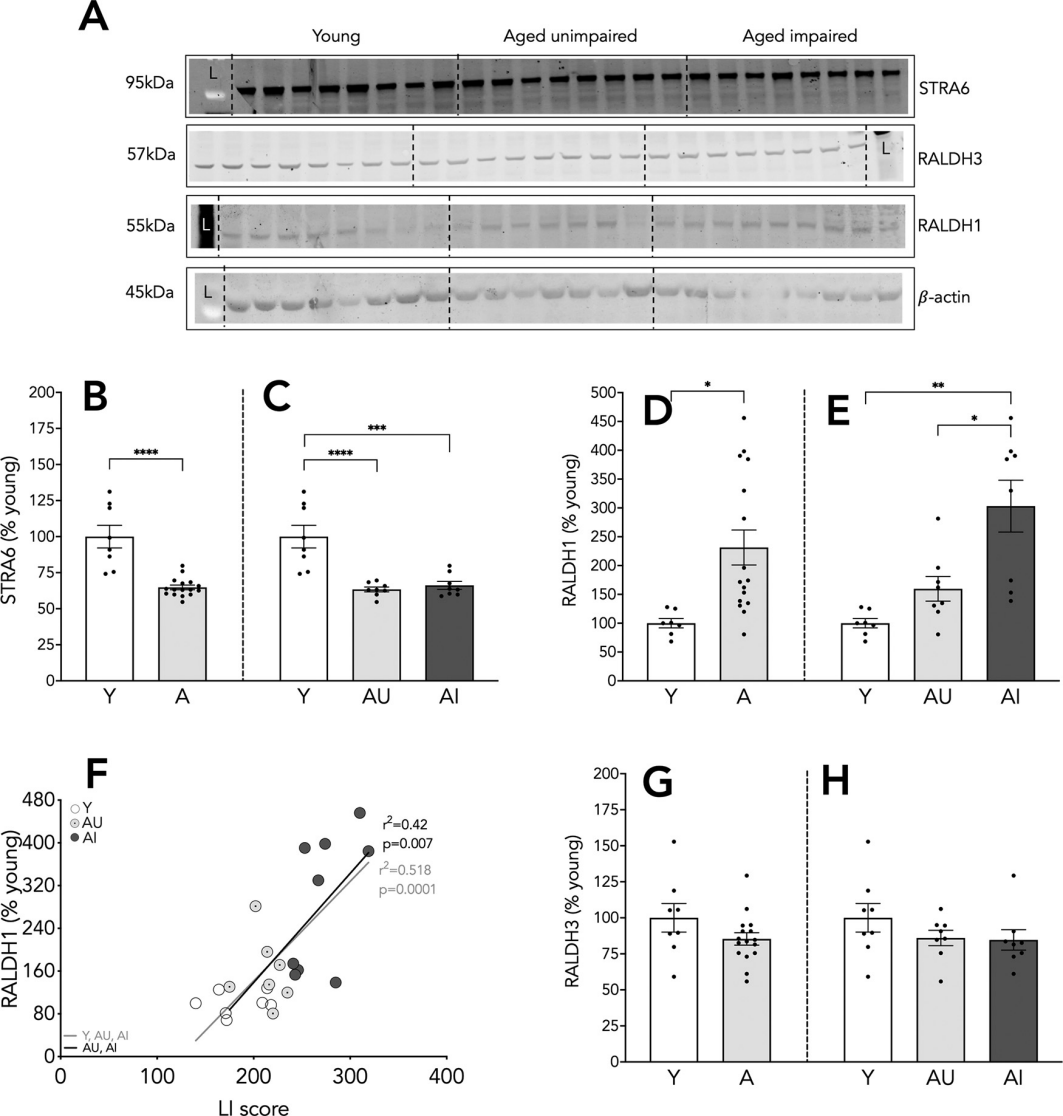
Protein expression of STRA6, RALDH1, and RALDH3 in the hippocampus. Representative blots for proteins of interest (***A***), expression relative to β-actin as a percentage of Y values, L stands for molecular ladder lane. Hippocampal expression of STRA6 (***B***, ***C***), RALDH1 (***D***, ***E***), and RALDH3 (***G***, ***H***). Correlation of protein expression of RALDH1 and LI scores (***F***). Results shown as bars with individual animal data plotted. Statistical analysis, unpaired two-tailed Student’s *t* test (***B***, ***D***, ***G***), one-way ANOVA, with Tukey’s multiple comparisons test (***C***, ***E***, ***H***), and linear regression (***F***; all animals, gray line; aged animals, black line); *p < 0.05, **p < 0.01, ***p < 0.001, ****p < 0.0001. Y *n* = 8 and aged *n* = 16 (AU *n* = 8, AI *n* = 8; ***B***, ***G***, ***H***); Y *n* = 7 and aged *n* = 16 (AU *n* = 7, AI *n* = 9; ***D–F***). Error bars represent SEM.

STRA6 is a cell surface receptor by which retinol enters the cell. We measured protein expression of this RA receptor in whole hippocampus from Y and aged animals. Levels of STRA6 were significantly lower in aged animals compared with Y (*t*_(22)_ = 6.00, *p* < 0.0001; Fig. 3*B*). This was true for aged rats without and with spatial memory impairment (*F*_(2,21)_ = 17.40, *p* < 0.0001; Y vs AU *p* < 0.0001; Y vs AI, *p* = 0.0002; Fig. 3*C*). Moreover, hippocampal STRA6 protein levels did not differ between the aged subgroups (Fig. 3*C*) or correlate with memory performance (Y and aged: *n* = 24, *r*^2^ = 0.0.121, *p* = 0.096; aged only: *n* = 16, *r*^2^ = 0.010 *p* = 0.148). These results indicate that, independent of cognitive outcome, hippocampal aging is associated with a reduction of the receptor allowing retinol cell entry.

### RA synthesis is increased in AI rats

The last step of RA metabolism is catalyzed by the RALDH enzymes. Here, we assessed the protein expression of two RALDH enzymes in whole hippocampal preparations, RALDH1 and RALDH3. Significantly higher levels of RALDH1 protein were found in aged rats relative to Y (*t*_(22)_ = 2.805, *p* = 0.011; Fig. 3*D*). This was the case only for AI, in which hippocampal RALDH1 expression was significantly higher than in both Y and AU animals (*F*_(2,20)_ = 9.314, *p* = 0.001; Y vs AI *p* = 0.001; AU vs AI *p* = 0.026; Fig. 3*E*). Notably, the expression of this enzyme correlated with LI scores among the aged animals such that rats with higher RALDH1 expression scored more poorly (i.e., higher LI scores; *n* = 16, *r*^2^ = 0.42, *p* = 0.007; Fig. 3*F*, black line). The correlation was similar when the Y and aged animals are considered together (*n* = 23, *r*^2^ = 0.518, *p* = 0.0001; Fig. 3*F*, gray line). RALDH3 protein expression in whole hippocampus was comparable in the Y and aged groups (*t*_(22)_ = 1.593, *p* = 0.125; Fig. 3*G*) and unrelated to cognitive status (*F*_(2,21)_ = 1.22, *p* = 0.315; Fig. 3*H*). In the aggregate, the results indicate that RALDH1 driven RA synthesis is potentially increased in the hippocampus of AI rats.

### AI animals display increased RA catabolism

**Figure 4.**
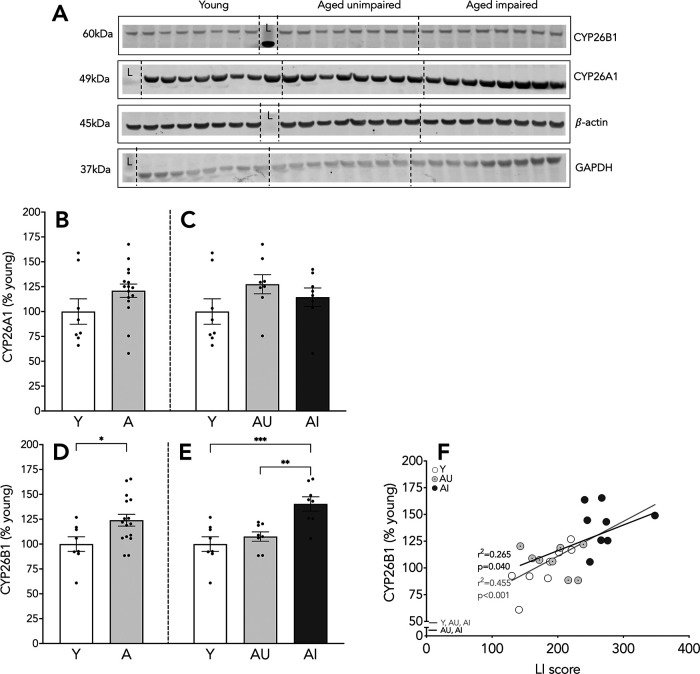
Protein expression of RA catabolizing enzymes in the hippocampus. Representative blots for proteins of interest (***A***), CYP26A1 expression relative to GAPDH, CYP26B1 expression relative to β-actin, L stands for molecular ladder lane. Hippocampal expression of CYP26A1 (***B***, ***C***) and CYP26B1 (***D***, ***E***). Correlation of CYP26B1 levels and LI scores (***F***). Results shown as bars with individual animal data plotted. Statistical analysis, unpaired two-tailed Student’s *t* test (***B***, ***D***), one-way ANOVA, with Tukey’s multiple comparisons test (***C***, ***E***), and linear regression (***F***; all animals, gray line; aged animals, black line); *p < 0.05, **p < 0.01, ***p < 0.001. Y *n* = 8 and aged *n* = 16 (AU *n* = 8, AI *n* = 8; ***B–F***). Error bars represent SEM.

RA is catabolized by the family of cytochrome p450 enzymes, CYP26. We measured the protein expression of CYP26A1 and CYP26B1 enzymes in the whole hippocampus preparations. CYP26A1 protein expression was largely overlapping between Y and aged rats (*t*_(22)_ = 1.609, *p* = 0.122; Fig. 4*B*) without and with spatial memory deficits (*F*_(2,21)_ = 1.651, *p* = 0.216; Fig. 4*C*). In contrast, we detected significantly higher CYP26B1 protein expression in the aged hippocampus (*t*_(22)_ = 2.428, *p* = 0.024; Fig. 4*D*). Interestingly, CYP26B1 levels were selectively increased in AI, differing from both Y and AU rats (*F*_(2,21)_ = 10.85, *p* = 0.0006; Y vs AI *p* = 0.0007; AU vs AI *p* = 0.005; Fig. 4*E*); levels in the latter groups were equivalent. Furthermore, there was a strong correlation between the expression of CYP26B1 and cognitive performance in the Y and aged rats (*n* = 24, *r*^2^ = 0.455, *p* = 0.0003; Fig. 4*F*, gray line), which was also robust among the aged animals alone (*n* = 16, *r*^2^ = 0.265, *p* = 0.040; Fig. 4*F*, black line), such that poor spatial memory was coupled with higher CYP26B1 enzyme levels. These results suggest that, although age-related increases in RA catabolism are selectively observed in aged animals with memory impairment, catabolic enzyme levels are coupled with spatial memory across the full range of individual differences observed in both Y and aged rats.

### Cellular and synaptosome RARα expression is increased in AI rats

**Figure 5.**
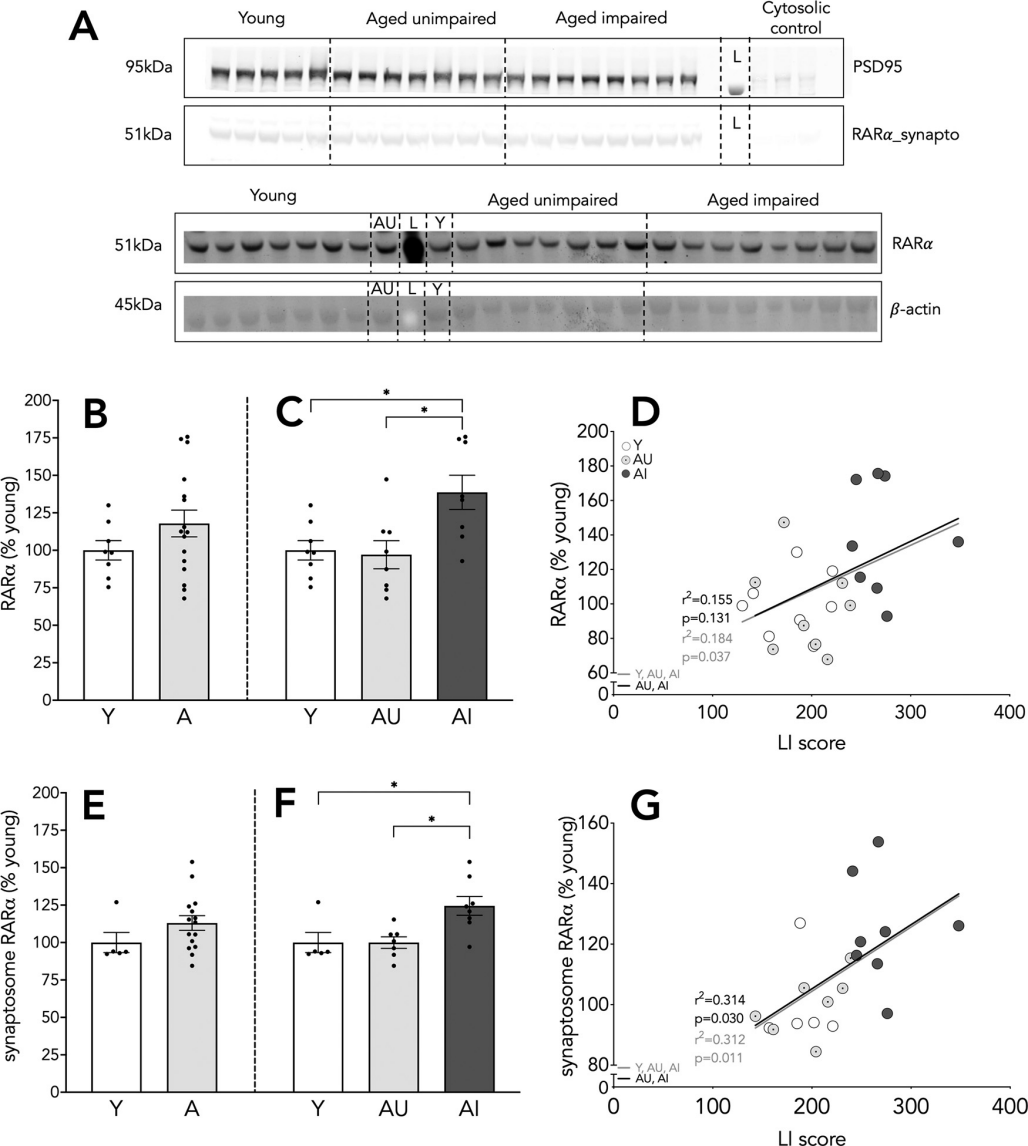
Cellular and dendritic RARα expression in the hippocampus. Representative blots for RARα (***A***), cellular RARα expression relative to β-actin (bottom two panels), and synaptosome RARα expression relative to PSD95 (top two panes); L stands for molecular ladder lane. Cytosolic fraction included as a confirmation of the fractionation. Hippocampal expression of RARα (***B***, ***C***). Correlation of RARα protein levels with LI scores (***D***). Synaptosome compartment RARα content (***E***, ***F***). Correlation of synaptosome RARα levels and LI scores (***G***). Results shown as bars with individual animal data plotted. Statistical analysis, unpaired two-tailed Student’s *t* test (***B***, ***E***), one-way ANOVA, with Tukey’s multiple comparisons test (***C***, ***F***), and linear regression (***D***, ***G***; all animals, gray line; aged animals, black line); C, cytosolic fraction; *p < 0.05. Y *n* = 8 and aged *n* = 16 (AU *n* = 8, AI *n* = 8; ***B–D***); Y *n* = 5 and aged *n* = 15 (AU *n* = 7, AI *n* = 8; ***E–G***). Error bars represent SEM.

RARα is one of six RA receptors but the only one that regulates homeostatic plasticity via non-nuclear action (Aoto et al., 2008; Yang et al., 2015). This receptor is involved in homeostatic synaptic scaling through its interaction with FMRP and GluR1. We measured RARα protein expression in whole hippocampus homogenates and found no differences in RARα expression between Y and aged animals (*t*_(22)_ = 1.321, *p* = 0.2; Fig. 5*B*). Interestingly, however, the expression of RARα was significantly elevated in AI animals in comparison with both Y and AU rats (*F*_(2,21)_ = 6.218, *p* = 0.008; Y vs AI *p* = 0.021, AU vs AI *p* = 0.013; Fig. 5*C*). In addition, cognitive scores correlated with the expression of RARα protein across the Y and aged rats (*n* = 24, *r*^2^ = 0.184, *p* = 0.037; Fig. 5*D*, gray line), such that animals with worse spatial memory showed higher RARα expression. Among the aged animals alone, water maze performance failed to correlate significantly with RARα protein expression (*n* = 16, *r*^2^ = 0.155, *p* = 0.131; Fig. 5*D*, black line). The strength of the association was nearly identical in both analyses, however, suggesting that the “aged only” result is less robust because of the decreased sample size and statistical power.

Non-genomic actions of RA signaling are mediated by RA receptors localized outside of the nucleus. We were specifically interested in the presence of RARα in the synaptosome preparations, because non-nuclear RARα acts as a mRNA granule containing GluR1 receptor mRNA. Therefore, we measured RARα protein levels in whole hippocampus synaptosome fractions. Similar to results for the whole cell lysates, we found no differences in RARα protein presence (*t*_(18)_ = 1.385, *p* = 0.18; Fig. 5*E*). However, AI animals displayed increased synaptosome RARα content relative to Y and AU rats (*F*_(2,17)_ = 6.561, *p* = 0.008; Y vs AI *p* = 0.026, AU vs AI *p* = 0.014; Fig. 5*F*), also similar to the pattern in whole lysates. Additionally, synaptosome RA receptor expression positively correlated with spatial memory performance among the Y and aged rats, with higher LI scores (i.e., poor memory) associated with increased RARα protein expression (*n* = 20, *r*^2^ = 0.312, *p* = 0.011; Fig. 5*G*, gray line). A similar positive correlation was also observed when the aged animals were considered alone in the analysis (*n* = 15, *r*^2^ = 0.314, *p* = 0.030; Fig. 5*G*, black line). These results indicate that RARα expression is increased selectively in aged animals with memory impairment in both whole cell and synaptosome preparations. Among the aged rats, levels of this receptor localized to the synaptosome compartment were coupled with individual differences in hippocampal memory.

### Hippocampal FMRP protein is increased in aged animals

**Figure 6.**
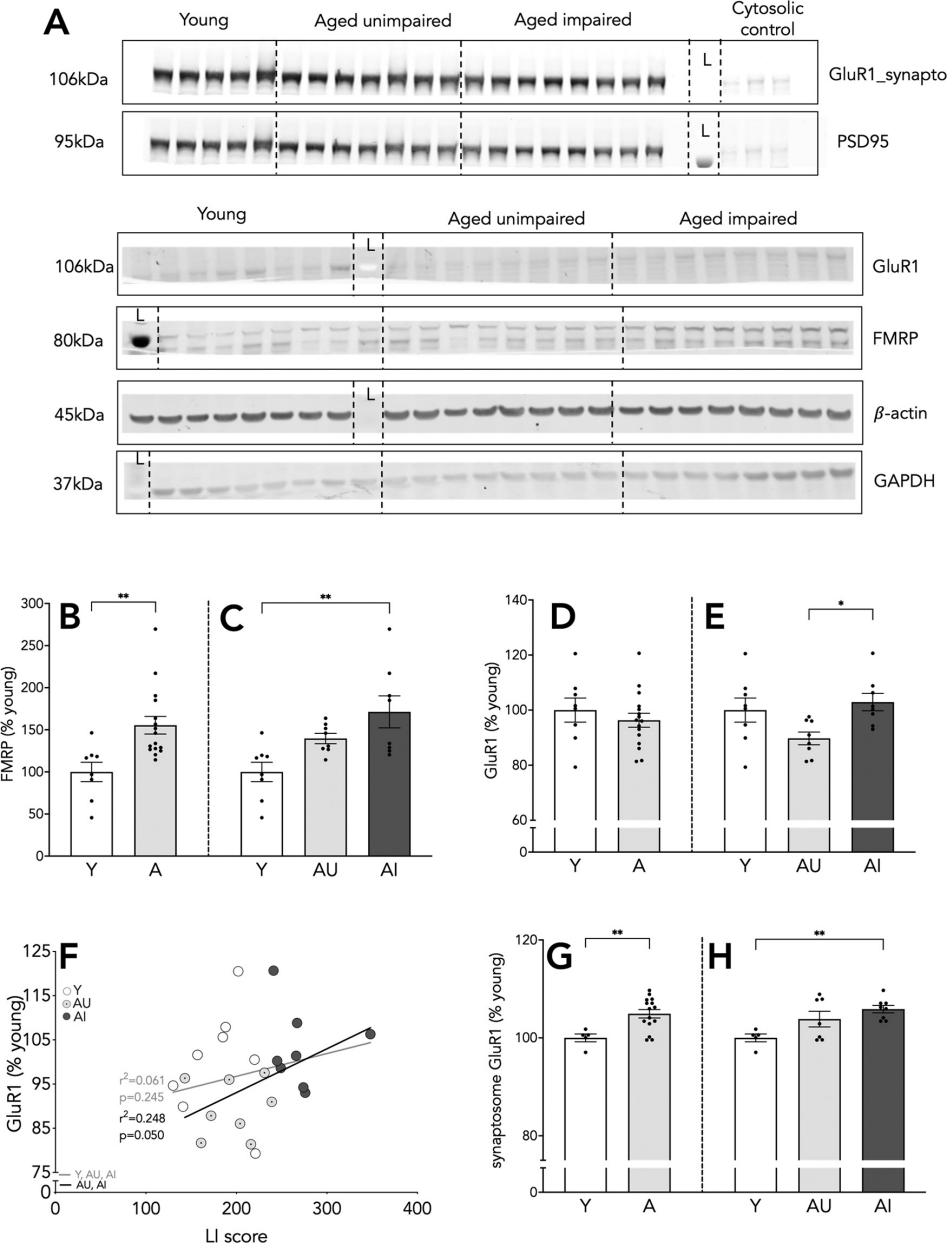
FMRP expression and cellular and dendritic expression of GluR1 in the hippocampus. Representative blots for proteins of interest (***A***), FMRP expression relative to GAPDH (bottom four panels); cellular GluR1 expression relative to β-actin, and synaptosome GluR1 levels relative to PSD95 (top two panels); L stands for molecular ladder lane. Cytosolic fraction included as confirmation of the fractionation. Hippocampal expression of FMRP (***B***, ***C***). Whole hippocampus (***D***, ***E***) and synaptosome GluR1 protein levels (***G***, ***H***). Correlation of cytosolic GluR1 protein expression with LI scores (***F***). Results shown as bars with individual animal data plotted. Statistical analysis, unpaired two-tailed Student’s *t* test (***B***, ***D***, ***G***), one-way ANOVA, with Tukey’s multiple comparisons test (***C***, ***E***, ***H***), and linear regression (***F***; all animals, gray line; aged animals, black line); C, cytosolic fraction; **p < 0.01. Y *n* = 8 and aged *n* = 16 (AU *n* = 8, AI *n* = 8; ***B–F***); Y *n* = 5 and aged *n* = 15 (AU *n* = 7, AI *n* = 8; ***G***, ***H***). Error bars represent SEM.

Next, we examined FMRP protein expression, which is required for the translation of GluR1 mRNA. We found significantly increased levels of FMRP protein in the aged hippocampus (*t*_(22)_ = 3.27, *p* = 0.0035; Fig. 6*B*). AI values were elevated relative to Y rats (*F*_(2,21)_ = 7.202, *p* = 0.004; Y vs AI *p* = 0.003; Fig. 6*C*), whereas results for AU were intermediate and failed to differ from either Y or AI (Fig. 6*C*). FMRP protein levels failed to correlate with LI scores, and overall, the data point to a general age-related increase in hippocampal FMRP.

### Cellular and synaptosome GluR1 levels are increased in aged animals with memory impairment

In the presence of FMRP, GluR1 mRNA is translated locally in a RARα regulated manner, enhancing AMPA receptor synaptic expression and strength. We measured GluR1 receptor protein levels in whole cell lysates and associated synaptosome preparations. No differences were observed in GluR1 expression between the Y and aged group (*t*_(22)_ = 0.776, *p* = 0.446; Fig. 6*D*). However, GluR1 protein was significantly increased in AI animals compared with AU (*F*_(2,21)_ = 4.161, *p* = 0.030, AU vs AI *p* = 0.031; Fig. 6*E*), although neither aged subgroup differed from Y. The correlation between GluR1 protein levels and spatial memory was not significant when the Y and aged animals were considered together (*n* = 24, *r*^2^ = 0.061, *p* = 0.245 Fig. 6*F*, gray line). However, a reliable correlation was observed among the aged animals (*n* = 16, *r*^2^ = 0.248, *p* = 0.050; Fig. 6*F*, black line), where poor spatial memory (i.e., high LI scores) was associated with higher hippocampal GluR1 levels.

In the synaptosome fraction, we found higher GluR1 protein expression in the aged rats than Y (*t*_(18)_ = 3.104, *p* = 0.006; Fig. 6*G*), and this effect appeared largely attributable to elevation among AI animals (*F*_(2,17)_ = 5.865, *p* = 0.012; Y vs AI *p* = 0.009; Fig. 6*H*). Although we found no direct correlation between spatial memory performance and synaptosome GluR1 levels, the results suggest that ionotropic glutamate expression in hippocampus, in both the cytosol and synaptosome, is predominantly increased in AI rats.

## Discussion

Research on the neurobiology of cognitive aging has traditionally focused on identifying differences between groups configured on the basis of chronological age. This approach, however, can obscure the increased individual variability that is a hallmark of aging in humans and animal models. Here, adopting a strategy validated in many previous studies (McQuail et al., 2018; Rapp et al., 1987, 2020), we explicitly capitalized on this variability to test whether changes in RA signaling are associated with differential cognitive outcomes in aging. The current evidence clearly documents that RA signaling in the hippocampus is disrupted across multiple levels of regulation in aged rats with memory deficits. Specifically, while levels of a key transporter of the substrate for RA (STRA6) were decreased in the aged hippocampus independent of cognitive status, changes in other components of the RA signaling pathway, including but not limited to synthesis and catabolism of RA (RALDH1, CYP26B1, RARα, FMRP, and GluR1), were selectively increased among aged animals with memory impairment. Levels of most of the affected RA signaling factors were reliably correlated with individual differences in spatial memory among aged rats. Although the specific mechanisms linking changes in RA signaling to disrupted memory-related plasticity remain to be determined, in the aggregate our results point to an overall increase in hippocampal RA signaling associated with age-related cognitive impairment. While previous studies have suggested that global RA decline leads to cognitive impairment (Etchamendy et al., 2001; Bonnet et al., 2008; Dumetz et al., 2020), our findings suggest that locally increased RA signaling is coupled with age-related cognitive decline, perhaps reflecting failed compensatory mechanisms. Alongside experimental design factors that might contribute to apparent discrepancies across studies, such as rat strain and diet, the current results highlight the importance of considering RA signaling in relation to individual variability in the cognitive outcome of aging. Nonetheless, a priority knowledge gap for future investigation is to explore the direction of causality, testing whether the observed effects of aging on RA signaling are a driver of, or response to, cognitive decline.

### Retinol availability in aged animals

The availability of retinol in the circulation is essential for the synthesis of RA. Circulating levels of retinol are dependent on dietary availability of vitamin A and are influenced by retinol storage in the liver, which is the largest storage site in the body (Napoli, 2012). The major pathway for retinol transport is through binding to RBP4/TTR complex (Li et al., 2014), and here, we used plasma RBP4 levels as a proxy for retinol content in the circulation. Animals in this study were maintained under identical conditions, ensuring that dietary retinol availability was constant and not the basis of the RBP4 differences seen between the aged groups.

Our results revealed significantly lower RBP4 plasma levels in AU animals compared with Y. This is consistent with recent findings in humans showing decreased plasma RBP4 content in aged individuals (Soo Lee et al., 2013; Kocełak et al., 2018). Interestingly, AI animals had RBP4 levels comparable to Y and significantly higher than AU rats. The liver and kidney prominently influence RBP4, because of their role in synthesis and excretion, respectively. Plasma triglyceride levels, which are indicative of liver function, were comparable across groups, suggesting that impaired liver function is unlikely to drive changes in RBP4. Renal function also affects circulating RBP4 and can be assessed by plasma creatinine levels, where high levels are indicative of kidney disease. Kocełak et al. (2018) found increased circulating RBP4 in patients with chronic kidney disease. That study concluded that plasma levels of this RBP predominantly reflect poor kidney function and are only modestly sensitive to aging. Here, we found no age-related change in this binding protein. Overall, plasma RBP4 in aged animals strongly correlated with LI scores, where low levels were detected in AU plasma. Together, this pattern of results raises the possibility that the selective decrease of plasma RBP4 seen in AU rats may be a component of an adaptive cascade, reducing retinol availability in blood, and providing a potential biomarker of resilient cognitive aging.

Some studies have reported an increase in RBP4 with obesity and insulin resistance (Esteve et al., 2009; Shajarian et al., 2015), whereas others have found no relationship (Ülgen et al., 2010; Kocełak et al., 2018). Aged rats in the present experiment did not display visible signs indicative of disrupted glucose regulation (e.g., increased water consumption and urination, or sharp weight gain), confirmed by normative circulating glucose levels. Additionally, plasma triglyceride content, a core component of the metabolic syndrome, was similar between groups. Creatinine levels further suggested that the rats were free of underlying renal disease that might influence RBP4 levels. Although the aged animals were significantly heavier and exhibited greater adiposity than Y, RBP4 levels were unrelated to body weight. Therefore, differences in plasma RBP4 among the aged rats were not secondary to weight gain or systemic metabolism change, and instead point to a potential RA influence on cognitive aging independent of metabolic aging.

### Global increase of RA metabolism in AI animals

A significant role of RA in memory is emerging, complementing reports that, in the hippocampus, RA is involved in homeostatic synaptic scaling (Aoto et al., 2008; Sarti et al., 2012; Hsu et al., 2019). Although there is evidence that retinol signaling is altered in the aged brain (Enderlin et al., 1997; Touyarot et al., 2013), the involvement of RA in cognitive aging has received limited attention.

Our findings establish that protein levels of STRA6 receptor, which plays an important role in the retinol transport across blood-tissue barriers (Kelly et al., 2016), are decreased in the aged hippocampus. In brain, expression of this receptor is regulated by the availability of vitamin A, and in tissues other than the eye, cytosolic retinol concentration is only partly regulated by STRA6 (Berry et al., 2013). In our model, age-related decline in hippocampal STRA6 expression does not appear to be a consequence of systemic change in retinol, as the results fail to parallel the observed changes in plasma RBP4 levels. Regardless of the mechanism, which remains to be determined, the reduction in STRA6 may reduce intracellular retinol availability. This is consistent with decreased retinoid brain levels in aged mice (Kelly et al., 2016).

We also examined the abundance of RA synthesizing and catabolizing enzymes in the hippocampus. Retinal dehydrogenase enzymes are responsible for the last step of RA synthesis. In the present experiments, RALDH1 expression was increased in aged animals, whereas RALDH3 levels were unchanged. Increased RALDH1 levels in the aged hippocampus contrast with reports of reduced retinol metabolism in other tissues (Etchamendy et al., 2003; Van Der Loo et al., 2004; Das et al., 2014) raising the possibility that the increase is a brain-specific response in aging. In the future it will be useful to extend the analysis to include the third RA synthesizing enzyme, RALDH2. The RA catabolic enzymes CYP26A1 and CYP26B1 are both expressed in the rat hippocampus (McCaffery and Simons, 2007; Stoney et al., 2016). CYP26A1 has greater catalytic activity for RA than CYP26B1 (Topletz et al., 2012), although CYP26B1 is more widely distributed in the brain (Stoney et al., 2016) and tightly regulates RA signaling during development (Abu-Abed et al., 2002). CYP26 gene expression is dynamically regulated by dietary retinol and RA from liver and extrahepatic tissues (Ray et al., 1997; Wang et al., 2002) While liver CYP26B1 is reportedly upregulated in aged subjects (Yamamoto et al., 2000), whether brain expression changes with age is unknown. Here, we found no difference in CYP26A1 between Y and aged animals, whereas CYP26B1 protein levels increased with age. In line with the observed increase in synthesizing enzyme, the predicted consequence of enhanced RA presence is a net increase in CYP26B1-mediated catabolism.

One of the non-genomic functions of RA is mediated by RARα, which undergoes active nuclear transport (Poon and Chen, 2008). This receptor is implicated in homeostatic synaptic scaling (Chen et al., 2014; Li et al., 2019), synaptic transmission in somatosensory cortex (Yee and Chen, 2016), and normal tactile sensory processing (Park et al., 2018). Here, we found significantly higher levels of RARα in AI rats, in both cytosolic and synaptosome fractions from hippocampus, suggesting that non-genomic RA action is affected in these animals. The presumed consequence is greater RARα availability for RA to bind to and release GluR1 mRNA, increasing availability for translation.

The translation of GluR1 mRNA critically depends on FMRP. This protein is not directly related to the RA signaling pathway, but it is an important mediator of RA’s downstream effects. An RNA-binding functional regulator, FMRP localizes to cytosolic membranes and the nucleus (Bostrom et al., 2016; Smidak et al., 2017), where it controls synaptic protein synthesis, modulating dendritic spine formation (Feng et al., 1997; Greenough et al., 2001; Weiler et al., 2004). Deficiency of FMRP leads to local protein synthesis-dependent endocytosis of GluR1 receptor (Nakamoto et al., 2007), and activation of GluRs influences dendritic FMRP localization (Antar, 2004). In contrast to previous reports (Singh et al., 2007; Smidak et al., 2017), our findings demonstrate modest but statistically reliable increases in hippocampal FMRP protein in aged rats, an effect predominantly attributable to AI rats. This increase, together with higher expression of GluR1, is positioned to potently influence excitatory neurotransmission in the hippocampus. Interestingly, pyramidal neurons in the hippocampal CA3 region of AI animals exhibit elevated firing rates (Wilson et al., 2005), and pharmacological treatments that reduce hyperactivity improve memory in both AI rats and MCI patients (Koh et al., 2010; Bakker et al., 2012). RA actions via RARα are also known to cause downscaling of synaptic inhibition by FMRP-dependent removal of synaptic GABA_A_ receptors (Sarti et al., 2013). It is possible, that altered GluR1 expression and network disinhibition lead to disrupted excitation/inhibition balance, which in turn may contribute to memory impairments observed in aged rats.

In conclusion, compelling evidence indicates that RA function extends well beyond metabolic control and includes regulation of memory-related synaptic plasticity. Here, we demonstrate that RA signaling in neurocognitive aging is affected at nearly all levels of regulation examined. We found a decrease in plasma RBP4 in aged animals without memory impairment. Net hippocampal RA signaling is likely increased in aged rats with cognitive impairment, reflecting in part greater synthesizing and catabolizing enzyme expression. Furthermore, we find increases in RARα, FMRP and GluR1 selectively in aged rats with memory impairments. These changes appear unrelated to metabolic aging, and instead most are specifically related to individual differences in the cognitive outcome of aging rather than chronological age. The importance of neuronal excitation/inhibition balance in relation to cognitive outcome has been highlighted in many studies and is the core of numerous neurologic diseases where altered RA signaling is implicated (Wołoszynowska-Fraser et al., 2020). Together, the current results lean in favor of increased RA signaling, potentially contributing to the excitation/inhibition imbalance that is prominently featured in age-related cognitive impairment. This work further extends the boundaries of RA function in brain, and specifically highlights the importance of considering aging effects in relation to individual variability in cognitive aging. Among potential future directions, a comprehensive account of RA signaling influences on neurocognitive aging will also require a parallel assessment of genomic pathway effects.

## Synthesis

Reviewing Editor: Alexander Soutschek, Ludwig-Maximilians-Universität München Decisions are customarily a result of the Reviewing Editor and the peer reviewers coming together and discussing their recommendations until a consensus is reached. When revisions are invited, a fact-based synthesis statement explaining their decision and outlining what is needed to prepare a revision will be listed below. The following reviewer(s) agreed to reveal their identity: Clementine Bosch-Bouju. Note: If this manuscript was transferred from JNeurosci and a decision was made to accept the manuscript without peer review, a brief statement to this effect will instead be what is listed below.

Reviewer 1

Well-characterized as a developmental morphogen, retinoic acid’s function in adult and aging brains is much less understood. This study by Woloszynowska-Fraser et al., examined hippocampal retinoic acid signaling in aged animals. Importantly, they grouped the aged cohort based on their spatial memory performances, thus allowing the establishment of correlation between cognitive performance and RA signaling levels. Interestingly, the authors found evidence that RA metabolism and signaling may be enhanced in aged animals with learning impairment. Additionally, hippocampal FMRP and GluA1 expressions were also found increased in aged animals. This is a descriptive and yet informative study as impaired RA signaling has been suggested to underly cognitive impairment in aging populations of human subjects. Given the previously established roles of RA in synaptic function, the paradoxical enhancement in RA signaling is rather intriguing. I have a few minor suggestions.

1. Given that RA regulates both synaptic excitation and inhibition, have the authors examined GABAa receptor expression levels in aged AU and AI animals?

2. The authors interpretation of the results is leaning toward increased RA signaling contributes to E/I imbalance and cognitive impairment in aged animals. However, since the data in this study is correlational, the authors perhaps should also consider the possibility that it is the other way around - age-dependent changes that lead to cognitive impairment also trigger compensatory increases in RA signaling (e.g. reduced neuronal activity in AI animal may drive RA synthesis and enhanced RA signaling, which leads to enhanced GluA1 synthesis as a homeostatic mechanism). Perhaps the authors can add these alternative interpretations to the discussion?

Reviewer 2

The manuscript by Wołoszynowska-Fraser et al., presents an interesting research, generally well conducted. The topic is of interest and this paper will definitely be of importance for the community investigating the role of retinoid signaling in brain function. The text is well written, the figures are clear and the literature is right. However, the manuscript exhibits some weaknesses, mainly related to the way the data are analyzed. I have some major and minor points that should improve the quality of the manuscript.

Major comments

Methods and results

• The learning index appears as a powerful statistical mean to tackle the diversity of memory impairments in aged animals. In your study, the learning index and the cut-off is the initial step in the paper to segregate aged rats between impaired and unimpaired, which is then used throughout the paper. However, details are missing concerning the calculation of the learning index and the cut-off (the reference cited is not open access). Please detail how the learning index was calculated, and how the value for the cut-off is chosen. Please also provide the learning curves of the animals, to help the reader to better visualize memory impairments for these animals.

• This research is focused on retinoic acid signaling which is dependent on vitamin A supply, as it is stated in the introduction of the manuscript. However, there is no mention of vitamin A supply for these animals. Therefore, the reader is missing critical details for its understanding of the study. Could you please detail in the method the diets of the animals: What was the amount of vitamin A supply during rats’ lifespan? What was the formulation of vitamin A source (retinyl palmitate, etc.)?

• Going with the previous point, only 1 proxy is presented in the manuscript for circulating retinol (plasma RBP4 concentration), is it possible to present another one?

• What is the rational for measuring RALDH1 levels in the hippocampus. It appears from the literature that RALDH1 is lowly expressed in the hippocampus, while RALDH2 and RALDH3 are expressed in the hippocampus. This very low expression of RALDH1 is visible in the figure 3A of the manuscript. Yet the variations between groups is up to 400% which may be exaggerated by the very low level of RALDH1 in the hippocampus. Please either present RALDH2 data, or justify why you are looking specifically at RALDH1 and what could be the rational for such an increase with aging.

Statistics

• In the first paragraph of the results, the text mentions a ‘substantial increased variability’ in aged rats for LI index. However, no quantification for this variability is provided. Could you please quantify this ‘increased variability’ (Figure 1A)? If possible, please use an appropriate statistical analysis that considers the different ‘n’ between the two groups (16 vs 32).

• The number of animals in Figure 1A (LI score) is higher than for correlations. Are they always the same rats that are used for subsequent analyses? If yes, could you please label in Figure 1A the animals that are used afterwards for correlations?

• Please correlate creatinine, glucose and TG with LI score as well. These analyses may reinforce your interpretation of the data on the interaction between metabolic status and memory impairments in aged rats.

• For western blot analyses, representing data as % of young is hiding part of the data. Could you please give the data simply normalized to the expression of control proteins (GAPDH or actin)? These data can appear in the extended data. This may be important for proteins that are lowly expressed.

Discussion

• The manuscript is investigating and discussing the non-genomic pathway of RA signaling to explain the relationship between RA signaling and memory impairment. However, the genomic pathway of RA signaling may also be involved. Could you please explain why you are focusing on non-genomic aspects?

• For some synaptic proteins, the expression is tightly regulated by learning processes. From the literature, are they evidence that GluR1 and/or FMRP can be modified by water-maze task itself? If such literature does exist, please discuss this point.

• As you are explaining in your discussion, your results are not exactly in accordance with previous literature. Do you have hypotheses to explain the differences between this work and previous papers? It may be related to the diet, the strain of the rats or other parameters. Discussing this in the manuscript may help the community to better understand the role of RA signaling in age-related memory impairments.

Minor

- What is the rational for having animals single housed?

- Please mention the number of animals for each analysis. It is sometimes written, but not everywhere.

- It is written ‘data not shown’ for the correlation between STRA6 and LI score. Please indicate the statistics in brackets instead.
